# Orofacial EMG in Congenital Multiple Cranial Neuropathies

**DOI:** 10.15844/pedneurbriefs-29-9-3

**Published:** 2015-09

**Authors:** Vamshi K. Rao

**Affiliations:** 1 Division of Neurology, Department of Pediatrics, University of Nebraska Medical Center and Children's Hospital and Medical Center, Omaha, NE

**Keywords:** Infant, Cranial Neuropathies, EMG

## Abstract

Investigators from Armand-Trousseau hospital and University of Paris studied 90 infants aged birth to 6 months with multiple cranial nerve involvement.

Investigators from Armand-Trousseau hospital and University of Paris studied 90 infants aged birth to 6 months with multiple cranial nerve involvement. Neurophysiologic studies using blink responses (BR’s) and electromyography (EMG) of the muscles of the face, tongue and soft palate were performed to investigate for orofacial function.

Neurogenic pattern was noted with involvement of facial nerve in 82/90 patients, abnormal BR’s in 40/63, pharyngeal plexus in 56/89 and hypoglossal nerve in 25/90. Poor outcome (presence or absence of neurological disabilities, need for respiratory assistance, gastrostomy and prolonged enteral feeding) and death was higher (P=0.02) where EMG identified >= 4 affected cranial nerves. There was however no significance associated with involvement of lower cranial nerves and poor outcome. [1]

COMMENTARY. Studies of cranial nerves in children using EMG is a time intense process involving a trained electromyographer and equally trained supportive staff. The investigators previously published studies where orofacial EMG’s were performed in children [2] for assessing brainstem involvement [3] and dysphagia in infants with facial malformations [4]. They combined the techniques to study all children referred to their center for orofacial dysfunction and observed outcome related to the number of cranial nerves involved.

Orofacial dysfunction is assumed to be of suprabulbar origin and although the cerebral cortex is more vulnerable to ischemia, neuropathological studies have shown brainstem involvement in birth asphyxia [5] and prenatal ischemia [6] . There are reports of hypoglossal involvement in children where periventricular leukomalacia or hemorrhagic infarction was thought to be the cause of dysphagia [7]. In fact, 14 patients of this study were thought to have a cortical vascular insult at birth with orofacial dysfunction but EMG studies showed face, tongue and soft palate neurogenic changes.

The study highlights that EMG’s are not only useful for peripheral nerves and the muscles they innervate, but can be employed for cranial nerve assessment. Localizations with respect to brainstem pathology can be inferred by testing multiple cranial nerve innervations in the facial and bulbar muscles. Neurophysiology is also helpful as clinical exam alone detected 3 times less facial nerve involvement compared to EMG, especially when the involvement was bilateral. EMG aided in diagnosis of lingual and pharyngeal abnormalities that were difficult to pick up clinically.

This study also underscores a poor outcome based on the number of cranial nerve involvement and not the cranial nerve type based on location in the brainstem. It is surprising that lower cranial nerves thought to induce a higher risk of airway compromise and aspiration, were not associated with poor outcome.
